# Ataxia Telangiectasia Mutated and Rad3 Related (ATR) Protein Kinase Inhibition Is Synthetically Lethal in XRCC1 Deficient Ovarian Cancer Cells

**DOI:** 10.1371/journal.pone.0057098

**Published:** 2013-02-25

**Authors:** Rebeka Sultana, Tarek Abdel-Fatah, Christina Perry, Paul Moseley, Nada Albarakti, Vivek Mohan, Claire Seedhouse, Stephen Chan, Srinivasan Madhusudan

**Affiliations:** 1 Laboratory of Molecular Oncology, Academic Unit of Oncology, School of Molecular Medical Sciences, University of Nottingham, Nottingham University Hospitals, Nottingham, United Kingdom; 2 Department of Clinical Oncology, Nottingham University Hospitals, Nottingham, United Kingdom; 3 Academic Haematology, School of Molecular Medical Sciences, University of Nottingham, Nottingham University Hospitals, Nottingham, United Kingdom; Dartmouth, United States of America

## Abstract

**Introduction:**

Ataxia telangiectasia mutated and Rad3 Related (ATR) protein kinase is a key sensor of single-stranded DNA associated with stalled replication forks and repair intermediates generated during DNA repair. XRCC1 is a critical enzyme in single strand break repair and base excision repair. XRCC1-LIG3 complex is also an important contributor to the ligation step of the nucleotide excision repair response.

**Methods:**

In the current study, we investigated synthetic lethality in XRCC1 deficient and XRCC1 proficient Chinese Hamster ovary (CHO) and human ovarian cancer cells using ATR inhibitors (NU6027). In addition, we also investigated the ability of ATR inhibitors to potentiate cisplatin cytotoxicity in XRCC1 deficient and XRCC1 proficient CHO and human cancer cells. Clonogenic assays, alkaline COMET assays, γH2AX immunocytochemistry, FACS for cell cycle as well as FITC-annexin V flow cytometric analysis were performed.

**Results:**

ATR inhibition is synthetically lethal in XRCC1 deficient cells as evidenced by increased cytotoxicity, accumulation of double strand DNA breaks, G2/M cell cycle arrest and increased apoptosis. Compared to cisplatin alone, combination of cisplatin and ATR inhibitor results in enhanced cytotoxicity in XRCC1 deficient cells compared to XRCC1 proficient cells.

**Conclusions:**

Our data provides evidence that ATR inhibition is suitable for synthetic lethality application and cisplatin chemopotentiation in XRCC1 deficient ovarian cancer cells.

## Introduction

Targeting DNA repair for synthetic lethality is an exciting new strategy for personalized therapy in ovarian cancer. DNA repair is essential for processing DNA damage induced by chemotherapy such as platinating agents (carboplatin, cisplatin) [1]. Intra-strand crosslink DNA adducts induced by platinating agents, if unrepaired, ultimately result in cell death [2], [3]. DNA intra-strand crosslinks are repaired predominantly by nucleotide excision repair (NER) in cells [4], [5]. Platinating agents can also generate oxygen free radicals that induce oxidative base damages that are processed by the DNA base excision repair (BER) pathway in cells [6], [7].

The XRCC1 (X-ray repair cross- complementing gene 1) protein is a critical factor in BER and single strand break repair pathway (SSBR). XRCC1-LIG3 complex is also an important contributor to the ligation step of the nucleotide excision repair (NER) response. XRCC1, a 70-kDa protein, has no known enzymatic activity (reviewed in [8], [9], [10]). XRCC1 functions as a molecular scaffold protein and coordinates DNA repair by interacting with several components of BER/SSBR such as PARP-1 [Poly(ADP-ribose)polymerases 1], DNA glycosylases, AP endonuclease (APE1) and others (reviewed in [8], [9], [10]). XRCC1 deficiency in cells lead to accumulation of DNA single strand breaks (SSBs), induce mutations and result in elevated levels of sister chromatid exchanges. XRCC1 deficiency in cell lines result in hypersensitivity to ionizing radiation and chemotherapy [9]. In human association studies, germline polymorphisms in XRCC1 may influence cancer risk [11], [12] and influence response to platinum based chemotherapy [13], [14], [15], [16]. In human ovarian cancer we have recently demonstrated that tumours frequently over-express XRCC1 (48%) and significantly associated with higher stage (p = 0.006), serous type tumours (p = 0.008), sub-optimal de-bulking (p = 0.004), a two fold increase of risk of death (p = 0.007) and progression (p<0.0001) [17]. In the multivariate analysis, XRCC1 expression was independently associated with survival in ovarian cancer patients [HR 2.3, p = 0.002]. XRCC1 negative tumours were associated with platinum sensitivity (p<0.0001). Pre-clinically we also confirmed that XRCC1 negative cells are hypersensitive to cisplatin compared to XRCC1 positive cells [17]. Hypersensitivity to cisplatin in XRCC1 negative cells was associated with accumulation of DNA strand breaks and G2/M cell cycle arrest [17]. Our data therefore suggests that XRCC1 is a promising biomarker in ovarian cancer.

Ataxia telangiectasia mutated and Rad3 Related (ATR) protein kinase is a key sensor of single-stranded DNA associated with stalled replication forks as well as generated during BER and double strand break repair as DNA repair intermediates. Activated ATR in turn phosphorylates a number of substrates involved in cell cycle regulation, DNA replication, DNA repair and apoptosis (reviewed in [18], [19], [20], [21], [22]). In preclinical studies, ATR inhibition may result in cytotoxic therapy sensitization [22], [23], [24]. Small molecule inhibitors of ATR are currently under development for therapeutic application in cancer [20], [21], [22].

The ability of PARP inhibitors to induce synthetic lethality in BRCA deficient ovarian cancers [25], [26], [27] suggests that additional factors within BER/SSBR may be suitable for such personalized approaches. XRCC1 is a critical factor in BER, SSBR and NER. ATR is a key sensor of SSBs. In the current study we have investigated and confirmed synthetic lethality in XRCC1 deficient cells treated with ATR inhibitors. Moreover, compared to cisplatin alone, combination of cisplatin and ATR inhibitor treatment results in enhanced cytotoxicity in XRCC1 deficient cells compared to XRCC1 proficient cells.

## Materials and Methods

### Compounds and Reagents

Small molecule ATR inhibitors NU6027 and VE-821 were purchased from Tocris Bioscience, UK and Tinib-Tools, Czech Republic respectively. The compounds were dissolved in 100% DMSO and stored at −20°C. Cisplatin (1 mg/ml) was obtained from the Department of Pharmacy, Nottingham University Hospitals, UK.

### Cell Lines and Culture

Previously well characterized Chinese hamster ovary (CHO) cells; CHO9 (Wild type), EM-C11 (XRCC1-mutant: C389Y substitution leading to XRCC1protein instability), EM-C12 (XRCC1-mutant: E98K substitution resulting in reduced XRCC1 protein integrity) [28] were provided by Professor Małgorzata Z. Zdzienicka, Department of Molecular Cell Genetics, Nicolaus-Copernicus University in Torun, Bydgoszcz 85-094, Poland. Cells were grown in Ham’s F-10 media (PAA, UK) [supplemented with 10% fetal bovine serum (FBS) (PAA,UK) and 1% penicillin/streptomycin]. EM9-V (XRCC1 mutant) and EM9 cells stably transfected with a human XRCC1 expression vector (EM9-XH cells) [29] were provided by Professor Keith Caldicott, Genome Damage and Stability Centre, University of Sussex, UK. Cells were grown in DMEM media (PAA, UK) [supplemented with 10% fetal bovine serum (FBS) (PAA,UK) and 1% penicillin/streptomycin]. Ovarian cancer cells OVCAR-3 and OVCAR-4 were grown in RPMI media (PAA, UK) [supplemented with 10% fetal bovine serum (FBS) (PAA,UK) and 1% penicillin/streptomycin].

### XRCC1 Knockdown Using siRNAs

Three XRCC1 siRNA constructs (sequences listed in Table 1) and one negative scrambled control and siRNA for Glyceraldehyde 3-phosphate dehydrogenase (GAPDH) (positive control) were used in these studies. The siRNA constructs were purchased from Ambion life technologies, UK. The transfection protocol was as described previously by Fan et.al [30]. Cells were plated in 6-well plates (2 ml medium/well without antibiotics). At 50% confluence, transfection was achieved using Lipofectamine TM 2000 (Invitrogen) according to the manufacturer’s protocol. Briefly, siRNA (100 pmol) and Lipofectamine (5 µl) were each separately mixed with 250 µl Opti- MEM1 (GIBCO/Invitrogen) without FBS. After 5 minute incubation at room temperature, the siRNA and Lipofectamine solutions were combined and incubated for another 20 min at room temperature. This mixture was then added to plated cells, cultured at 37°C overnight and the medium was later replaced with fresh medium plus penicillin/streptomycin (1%). When the cells attained 100% confluence, they were trypsinized and subsequently transferred into 75 cm^2^ flasks for continued growth and/or treatment. XRCC1 Knockdown was evaluated by western blotting at various time points after transfection (days 3, 5 and 7).

**Table 1:** XRCC1 siRNA constructs.

siRNA	Sequence
XRCC1_1 siRNA (ID s14940)	5′GGCAGACACUUACCGAAAAtt 3′-sense sequence3′ttCCGUCUGUGAAUGGCUUUU5′-antisense sequence
XRCC1_2 siRNA (ID s14941)	5′GGCAAGCACUUCUUUCUUUtt 3′-sense sequence3′tcCCGUUCGUGAAGAAAGAAA5′-antisense sequence
XRCC1_3 siRNA (ID s14942)	5′GCUUGAGUUUUGUACGGUUtt 3′-sense sequence3′acCGAACUCAAAACAUGCCAA5′ –antisense sequence

### Western Blot Analysis

Protein samples were prepared by lysing cells in RIPA buffer (20 mM Tris, 150 mM Nacl, 1% Nonidet p-40, 0.5% sodium deoxycholate, 1 mMEDTA, 0.1% SDS) containing protease inhibitor (Sigma) and phosphatase inhibitor cocktail 2 and 3 (Sigma) and then taken to western blot analyses as described previously [29]. Primary antibody were a mouse anti -XRCC1 (Thermo Fisher Scientific, Waltham, MA, USA) and rabbit anti-ATR (Novus Biologicals, USA). HRP conjugate secondary antibody were a rabbit anti-mouse and goat anti-rabbit respectively (Dako, Glostrup, Denmark).

### Clonogenic Survival Assay

Two hundred cells per well were seeded in six-well plates. Cells were allowed to adhere for 4 hours. NU6027 or VE-821 were added at the indicated concentrations and the plates were left in the incubator for 10 days for CHO cells. For siRNA transfected OVCAR-3 and OVCAR-4 cells, three days after transfection, NU6027 or VE-821 was added at indicated concentrations and the plates were left in the incubator for 14 days. For cisplatin and ATR inhibitor combination studies, cells were initially treated with cisplatin for 16 hours and then gently washed twice with 1X phosphate buffered saline and incubated in fresh media with or without NU6027 (4 µM for CHO cells and 6 µM for OVCAR-3 cells) for 10 days (CH cells) or 14 days (human cancer cells). After incubation, the media was discarded, fixed (with methanol and acetic acid mixture) stained with crystal violet and counted. Surviving Fraction = [No. of colonies formed/(No. of cells seeded x Plating efficiency)]. All clonogenic assays were done in triplicate.

### Alkaline COMET Assay

This assay was performed as described previously [31]. Briefly, sub-confluent cells were exposed to NU6027 (4 µM). At 24 hours, cells were extracted and alkaline comet assays were performed. Alkali electrophoresis buffer consisted of 200 mM NaOH, 1 mM EDTA and pH 13. The slides were then stained with SYBR® green (1∶10,000 dilution) (Molecular Probes) for 10 minutes and images were visualized under a rhodamine filter with an Olympus BX40 microscope. The comets were analysed using Comet Assay III image analysis software (Perceptive Instruments, Suffolk, UK). A total of 200 comet images were evaluated for olive tail moment.

### γH2AX Immunocytochemistry

Cells were treated for 48 hours with NU6027 (4 µM for CHO cells and 6 µM for OVCAR-3 cells) and the assay was performed as described previously [31]. For cisplatin and NU6027 combination studies, initially the cells were treated for 16 hours with cisplatin (1.5 µM for CHO cells and 1 µM for OVCAR-3 or OVCAR-4 cells) and then gently washed twice with 1X phosphate buffered saline and incubated in fresh media with or without NU6027 (4 µM for CHO cells and 6 µM for OVCAR-3 or OVCAR-4 cells) and the assay was performed as above. The frequencies of cells containing γH2AX foci were determined in 100 cells per slide in three separate experiments. Nuclei containing more than six γH2AX foci were considered positive.

### Flow Cytometric Analyses (FACS) for Cell Cycle Progression

Cells were treated for 24 hour with NU6027 (4 µM for CHO cells and 6 µM for OVCAR-3 or OVCAR-4 cells). Cells were later collected by trypsinization and centrifugation (1000 rpm for 5 minutes) and FACS analyses were performed as described previously [31].

### Apoptosis Detection by FITC-annexin V Flow Cytometric Analysis

Cells were treated for 48 hours with NU6027 (4 µM for CHO cells and 6 µM for OVCAR-3 or OVCAR-4 cells). Cells were later collected by trypsinization and centrifugation (1000 rpm for 5 minutes) and were washed twice with cold PBS and then re-suspended cells in 1X Binding buffer at a concentration of 1×10^6^ cells/ml. Then 100 µl of the solution (1×10^5^ cells) was transferred to a 5 ml culture tube and 5 µl of FITC Annexin V and 5 µl PI were added. The cells were then gently vortex and incubated for 15 minutes at room temperature (25°C) in the dark. After the incubation, 400 µl of binding buffer was added to each tube and was analyzed by flow cytometry within 1 hour. For cisplatin and NU6027 combination studies, the cells were initially treated for 16 hours with cisplatin (1.5 µM for CHO cells and 1 µM for OVCAR-3 or OVCAR-4 cells) and then gently washed twice with 1X phosphate buffered saline and incubated in fresh media with or without NU6027 (4 µM for CHO cells and 6 µM for OVCAR-3 or OVCAR-4 cells) and the assay was performed as described previously. Data was analysed using FlowJo7.6.1 software.

### Evaluation of Drug Interaction (Combination Index)

To investigate synergistic and additive activity, combination index was calculated as described previously [30]. Dose-response curves for cisplatin or NU6027 alone were first generated. The effect of the combined treatment was then analysed for the combination of drug A (cisplatin) and B (NU6027), by applying the following equation: Ac/Ae+Bc/Be = D, where Ac and Bc correspond to the concentrations of drugs used in the combination treatment, and Ae and Be corresponds to the concentrations of drugs able to, by themselves, produce the same magnitude of effect. If D (combination index) is <1 the effect of the combination is synergistic, whereas if D = 1 or D is >1 the effect is additive or antagonistic respectively [32].

## Results

### Synthetic Lethality

To evaluate synthetic lethality pre-clinically, a panel of XRCC1 deficient and XRCC1 proficient Chinese Hamster Ovary and human ovarian cancer cell lines were treated with small molecule inhibitors of ATR (NU6027 and VE-821).

#### Chinese Hamster Ovary (CHO) cells

**Figure 1:**
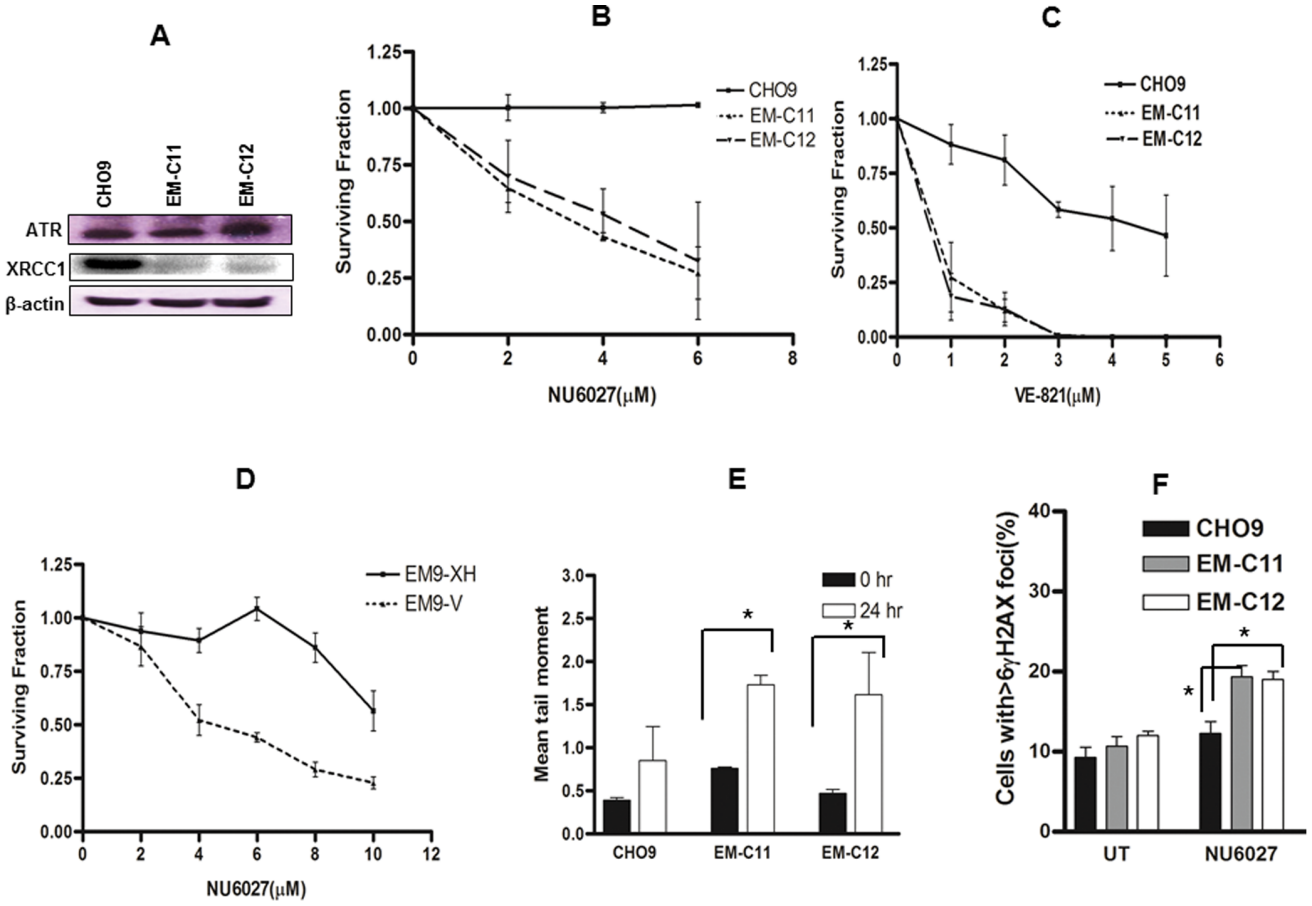
Western blot analysis in chinese hamster (CH) cells (CHO9, EM-C11, EM-C12) (A). Clonogenic survival assays for CH cells treated with NU6027 **(B)** and VE-821 **(C)** at indicated concentrations (see methods for details). **D.** Clonogenic survival assays for EM9-V and EM9-XH cells treated with NU6027. **E.** Alkaline COMET assay in CH cells treated with NU6027. EM-C11 and EM-C12 demonstrated a higher mean tail moment compared to CHO9 cells. **F.** EM-C11 and EM-C12 cells accumulate significantly higher γH2AX foci compared to CHO9 cells upon NU6027 treatment. Data represent mean values ±SEM (n = 6). Results were analysed using Students t-test. * p<0.05.

CHO9 (Wild type), EM-C11 (XRCC1 deficient) and EM-C12 (XRCC1 deficient) were investigated in clonogenic survival assays. We initially evaluated XRCC1 and ATR expression status in CHO9, EM-C11 and EM-C12 cells. Western blot analysis in Figure 1A demonstrates that EM-C11 and EM-C12 have no measurable XRCC1 protein expression compared to CHO9. EM-C11, EM-C12 and CHO9 are proficient in ATR expression. Figure 1B shows that EM-C11 and EM-C12 cells are sensitive to NU6027 treatment compared to CHO9 cells. Similarly, EM-C11 and EM-C12 cells are also sensitive to VE-821 compared to CHO9 cells (Figure 1C). To investigate if sensitivity of XRCC1 deficient cells to the ATR inhibitors can be corrected by expression of wild-type XRCC1 protein in XRCC1 deficient CHO cells, we performed clonogenic assays in EM9-V (XRCC1 mutant) and EM9-XH (cells stably transfected with a human XRCC1 expression vector). Figure 1D demonstrates that EM9-V cells are sensitive to NU6027 compared to EM9-XH.

We then conducted functional analysis in cells. ATR inhibition leads to DNA single strand break (SSB) accumulation. Therefore Alkaline COMET assay was performed. Figure 1E summarizes the results for CHO9, EM-C11 and EM-C12 cells treated with 4 µM of NU6027. Compared to pre-treatment samples, after 24 hours of exposure to ATR inhibitor, EM-C11 and EM-C12 cells demonstrate a significantly higher mean tail moment compared to CHO9 (p<0.01). The data confirms SSB accumulation following ATR inhibition in XRCC1 deficient cells.

DNA double strand breaks (DSBs) induce phosphorylation of H2AX at serine 139 (γH2AX). Accumulation of γH2AX foci in the nucleus is a marker of DSBs. Therefore, γH2AX immunocytochemistry was performed in EM-C11, EM-C12 and CHO9 cells treated with 4 µΜ of NU6027. Nuclei containing more than six γH2AX foci were considered positive. γH2AX immunocytochemistry confirmed that XRCC1 deficient EM-C11 and EM-C12 cells accumulated more γH2AX foci at 48 hours (p = 0.02 and p = 0.05) compared to CHO9 cells (Figures 1D).

**Figure 2:**
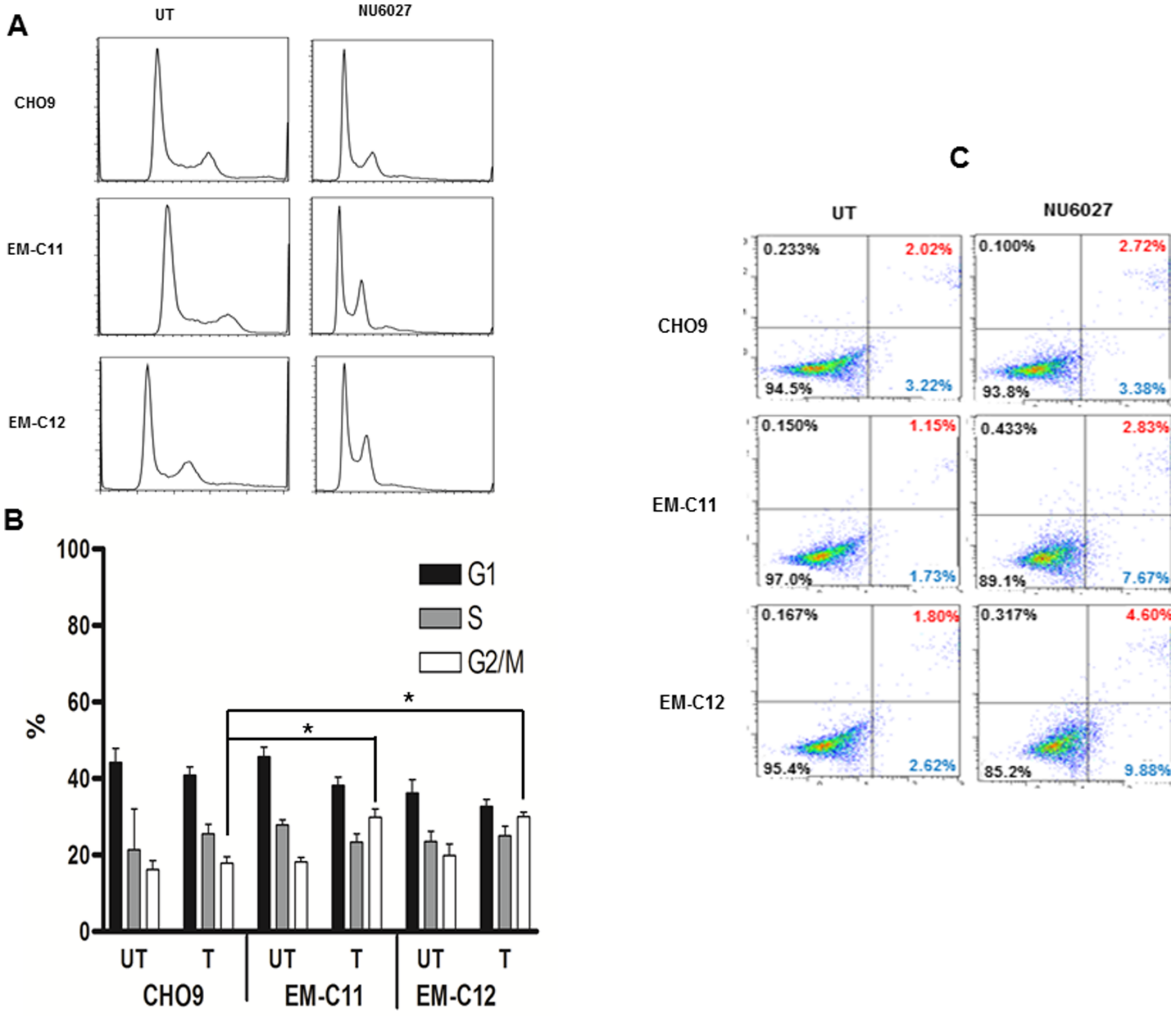
FACS read out in CH cells treated with 24 hours of NU6027 is shown here (A). **B.** Quantification of various phases of the cell cycle is shown for CH cell treated with NU6027. Data represent mean values ±SEM (n = 6). Results were analysed using Students t-test. * p<0.05. **C.** FITC-Annexin V apoptosis assay is shown here. The proportion of cells in early phase apoptosis is higher in XRCC1 deficient cells treated with NU6027 compared to wild type cells.

Accumulation of DSBs may delay cell cycle progression. FACS analyses were therefore performed in EM-C11, EM-C12 and CHO9 cells treated with NU6027 (4 µM). Cell cycle progression was evaluated and compared to control samples. At 24 hours, EM-C11 and EM-C12 were shown to be arrested in G2/M phase of the cell cycle (p = 0.01 and p = 0.03) compared to CHO9 cells (Figure 2A and 2B).

Accumulation of DSBs, if unrepaired, induces apoptosis in cells. Therefore, FITC-annexin V flow cytometric analysis was conducted to quantify apoptosis in cells treated with 4 µM of NU6027 and apoptotic cells quantified at 48 hours. In EM-C11 cells the proportion of cell in early apoptosis increased to 7.5% after 48 hours treatment with NU6027 compared to 1.73% in untreated cells. Similarly in EM-C12 cells, percentage of early apoptotic cells increased from 2.62% to 9.88%. On the other hand in CHO9 cells, there was no change in the percentage of early apoptotic cells (3.22% in untreated and 3.38% after 48 hours of NU6027 treatment (Figure 2C).

The data presented in Chinese Hamster cells suggests that XRCC1 deficient cells are sensitive to ATR inhibitors. ATR inhibition leads to increased DSB accumulation, G2/M cell cycle arrest and apoptosis. This suggests a synthetic lethality relationship between XRCC1 and ATR. To confirm this data further we investigated in human ovarian cancer cell lines.

#### Human ovarian cancer cells

**Figure 3:**
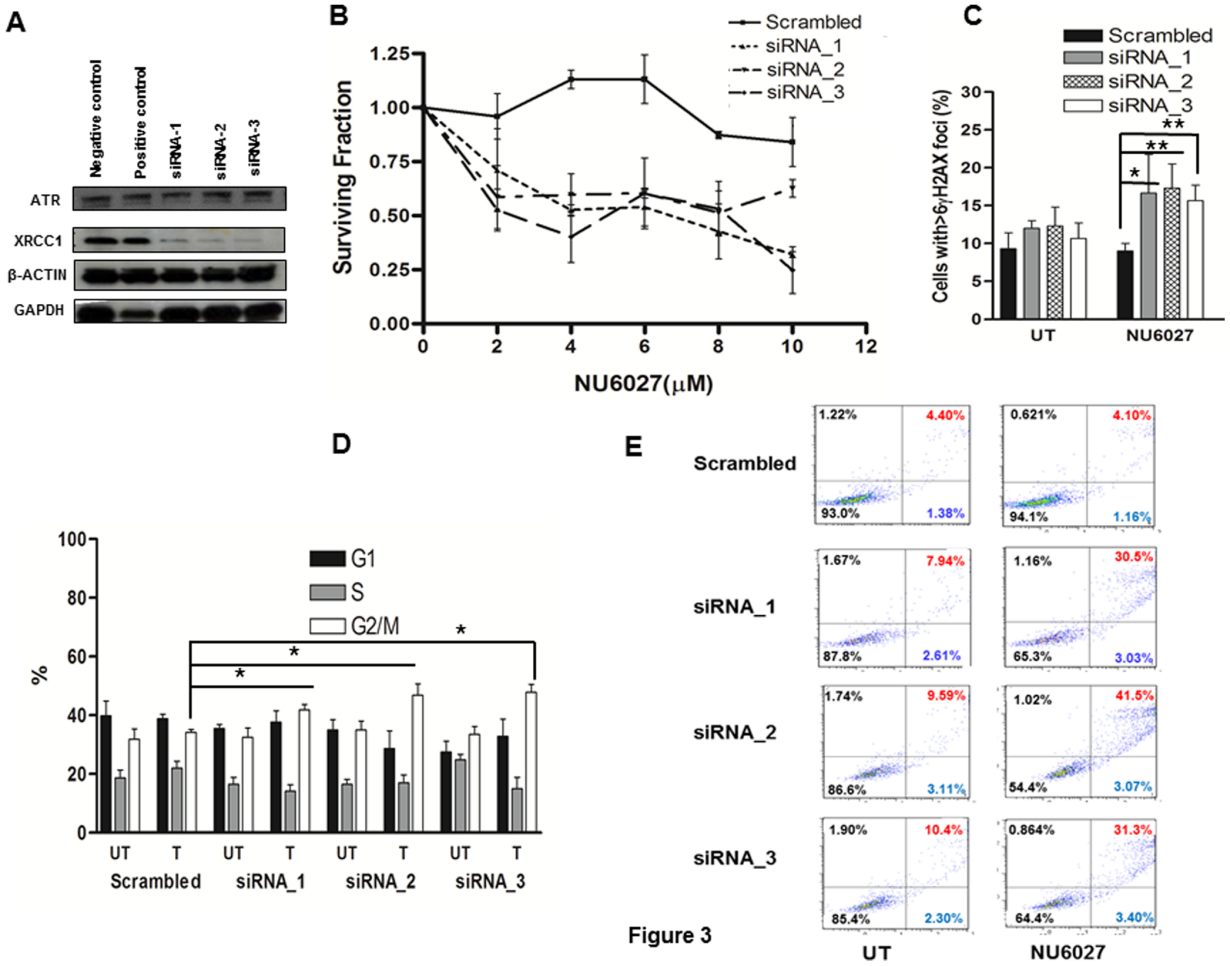
Western blot analysis in siRNA transfected OVCAR-3 cells is shown here (A). **B.** Clonogenic survival assays for siRNA transfected OVCAR-3 cells treated with NU6027 at indicated concentrations is shown here (see methods for details). **C.** XRCC1 deficient cells accumulate significantly higher γH2AX foci compared to scrambled control cells upon NU6027 treatment. Data represent mean values ±SEM (n = 6). Results were analysed using Students t-test. * p<0.05, ** p<0.01. **D.** Quantification of various phases of the cell cycle is shown for siRNA transfected OVCAR-3 cell treated with NU6027 is shown here. Data represent mean values ±SEM (n = 3). Results were analysed using Students t-test. * p<0.05. **E.** FITC-Annexin V apoptosis assay for siRNA transfected OVCAR-3 cells is shown here. The proportion of cells in late phase apoptosis is higher in XRCC1 deficient cells treated with NU6027 compared to scrambled control cells.

We generated XRCC1 knockdown human ovarian cancer cell lines using three siRNA constructs (Table 1). After transfection, cell lysates were sampled on days 3, 5 and 7 for XRCC1 knock down by western blot analysis. Figure 3A confirms that all three constructs (siRNA-1, siRNA-2, siRNA-3) induce efficient knockdown (more than 80%) of XRCC1 in OVCAR-3 cells on day 3 compared to scrambled negative control and GAPDH positive control. In clonogenic survival assays, NU6027 treatment reduced survival in XRCC1 deficient cells compared to proficient cells (Figure 3B). Similar results were also seen in OVCAR-4 cells (Figure S1 A). γH2AX immunocytochemistry confirmed that XRCC1 deficient cells accumulated more γH2AX foci at 48 hours (p = 0.05, p = 0.01 and p = 0.007 respectively) compared to scrambled control (Figure 3C). Moreover, at 24 hours, XRCC1 deficient cells were shown to be arrested in G2/M phase of the cell cycle (p = 0.05, p = 0.04 and p = 0.05) compared to CHO9 cells (Figure 3D). In XRCC1 deficient cells the proportion of cells in apoptosis increased substantially compared to scrambled controls upon NU6027 treatment (Figure 3E).

Taken together, the data from CHO cells and human cell lines provide convincing evidence that ATR inhibitors induce synthetic lethality in XRCC1 deficient cells.

### Cisplatin Chemopotentiation

**Figure 4:**
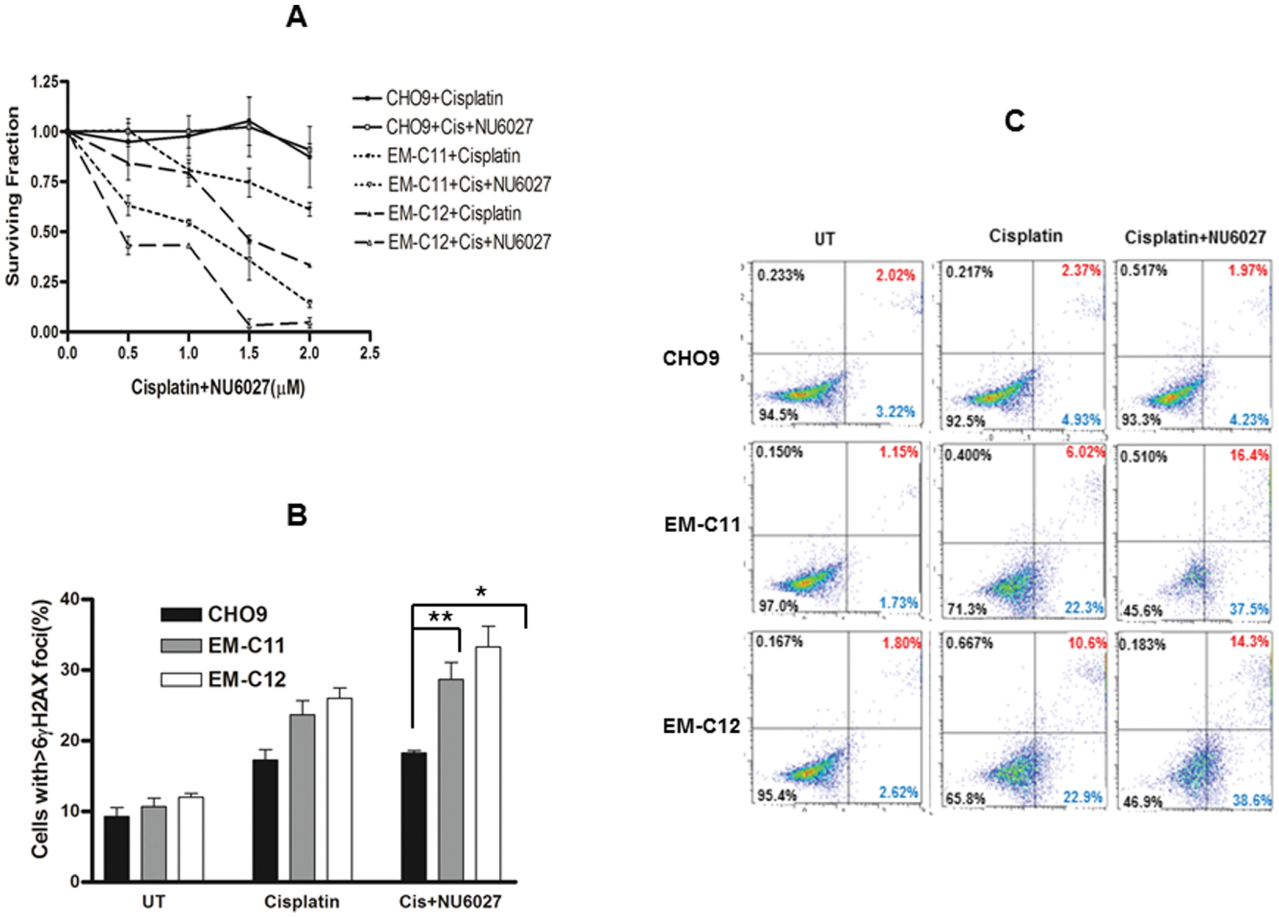
Clonogenic survival assays for CH cells treated with cisplatin alone or in combination with NU6027 is shown here (A). X-axis designates increasing concentration of cisplatin only. NU6027 was fixed at 4 µM. **B.** XRCC1 deficient CH cells accumulate significantly higher γH2AX foci compared to XRCC1 proficient CH cells upon cisplatin treatment alone or a combination of cisplatin and NU6027. Data represent mean values ±SEM (n = 6). Results were analysed using Students t-test. * p<0.05, ** p<0.01. **C.** FITC-Annexin V apoptosis assay is shown here. The proportion of cells in early phase as well as late phase apoptosis is higher in XRCC1 deficient cells treated with cisplatin alone or a combination of cisplatin and NU6027 compared to wild type cells.

We have previously demonstrated that XRCC1 deficient cells are sensitive to cisplatin [17]. In the current study, we first confirmed this observation in XRCC1 deficient EM-C11 and EM-C12 cells compared to CHO9 wild type cells (Figure 4A). We then evaluated combination strategies. The cytotoxicity of cisplatin was enhanced by NU6027 in XRCC1 deficient CH cells compared to XRCC1 proficient cells. In order to evaluate the interaction between NU6027 and cisplatin, combination index was calculated as described previously [32]. Cells were cultured in the presence of increasing doses of cisplatin (range 0.5–3 µM) in combination with a concentration of NU6027 able to induce a 50% growth inhibition. NU6027 potentiated the cytotoxic effect of cisplatin on XRCC1 deficient cells (Table 2). If combination index (D) is <1 the effect of the combination is synergistic, whereas if D = 1 or D is >1 the effect is additive or antagonistic respectively [32]. In the current study, the combination index was one in EM-C11 and EM-C12 cells. We concluded that enhancement of cisplatin cytoxicity by NU6027 in CHO cells was additive rather than synergistic. We then proceeded to conduct functional analyses in cells. Cisplatin alone treatment increased DSB accumulation in XRCC1 deficient cells which was further increased by NU6027 (p = 0.01 and p = 0.02) (Figure 4B). The DSB accumulation seen in XRCC1 deficient cells was also associated with accumulation of apoptotic cells as shown in Figure 4C.

**Table 2:** Effect of NU6027 and Cisplatin in Chinese hamster ovary and human ovarian cancer cells.

Cell lines		NU6027 (µM;Ac)	Cisplatin (µM;Bc)	NU6027 (µM;Ae)	Cisplatin (µM;Be)	D
**CHO**	EM-C11	4	1.5	8	3	1
	EM-C12	4	1.5	10	2.5	1
**OVCAR-3**	siRNA_1	6	1	10	2.5	1
	siRNA_2	6	1	12	2	1
	SiRNA_3	6	1	10	3	0.93
**OVCAR-4**	siRNA_1	6	1	9	3	0.99
	siRNA_2	6	1	10	2.5	1
	SiRNA_3	6	1	10	2.5	1

Ac and Bc, concentrations of drugs used in the combination treatment; Ae and Be concentrations of NU6027 alone and cisplatin alone respectively that produce a similar magnitude of effect; D (combination index.).

**Figure 5:**
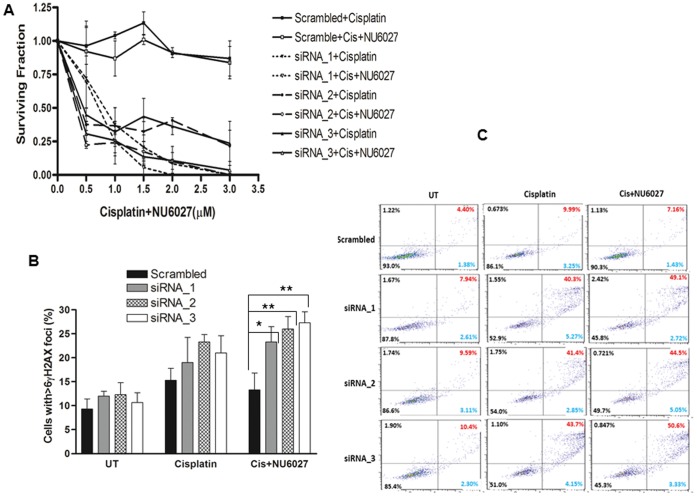
Clonogenic survival assays for siRNA transfected OVCAR-3 cells treated with cisplatin alone or in combination with NU6027 is shown here (A). **B.** XRCC1 deficient cells accumulate significantly higher γH2AX foci compared to XRCC1 proficient cells upon cisplatin treatment alone or a combination of cisplatin and NU6027. Data represent mean values ±SEM (n = 6). Results were analysed using Students t-test. * p<0.05, ** p<0.01. **C.** FITC-Annexin V apoptosis assay is shown here. The proportion of cells in late phase apoptosis is higher in XRCC1 deficient cells treated with cisplatin alone or a combination of cisplatin and NU6027 compared to wild type cells.

We then conducted similar studies in siRNA transfected human OVCAR-3 or OVCAR-4 cells. Similar to the results seen in CH cells, XRCC1 deficient OVCAR-3 or OVCAR-4 cells were sensitive to cisplatin. NU6027 enhanced cytotoxicity of cisplatin in XRCC1 deficient OVCAR-3 cells compared to XRCC1 proficient cells (Figure 5A). Similar results were also seen in OVCAR-4 cells (Figure S1 B). Combination index studies (Table 2) demonstrated that in most cells was one, except for OVCAR-3 cells treated with Si RNA_3 (D = 0.93) and OVCAR-4 cells SiRNA_1 (D = 0.99). Taken together, we concluded that human ovarian cancer cells the potentiating effect is likely to be additive. Cisplatin alone treatment increased DSB accumulation in cells which was further increased by NU6027 (p = 0.02, p = 0.007 and p = 004) (Figure 5B). The DSB accumulation seen in XRCC1 deficient cells was associated with accumulation of substantial apoptotic cells as shown in Figure 5C.

The data presented here not only provides further evidence that XRCC1 deficient cells are sensitive to cisplatin chemotherapy but also suggests that ATR inhibition additively enhances cisplatin toxicity in XRCC1 deficient cells compared to XRCC1 proficient cells.

## Conclusions

ATR protein kinase is a key sensor of single-stranded DNA associated with stalled replication forks and repair intermediates generated during BER and DSB repair. ATR activation regulates several cellular processes including cell cycle regulation, DNA replication, DNA repair and apoptosis. XRCC1 is essential for BER and SSBR and contributes to the ligation step of the NER response. We hypothesised that ATR inhibition could be synthetically lethal in XRCC1 deficient cells.

In the current study we have confirmed that ATR inhibitors are synthetically lethal in XRCC1 deficient cells. We have concluded synthetic lethality for the following reasons. First, CHO cells as well human cancer cells deficient in XRCC1 were highly sensitive to ATR inhibitors. Second, functional analyses demonstrated that ATR inhibition in XRCC1 deficient cells led to an accumulation of DNA DSBs, G2/M cell cycle arrest and increased apoptosis. This data is consistent with a study by Peasland et al [22] who demonstrated that NU6027 is synthetically lethal in cell treated with a PARP inhibitor that blocks BER. Moreover, the authors also demonstrated that EM9 chinese hamster cells lacking XRCC1 are also sensitive to NU6027 [22]. The data, including ours, therefore provides compelling evidence that ATR inhibition is synthetically lethal in BER deficient cells. We present a working model for ATR inhibition as a synthetic lethality strategy in XRCC1 deficient cells. In brief, ATR inhibition leads to SSB accumulation. Cells deficient in XRCC1 are unable to process SSBs which are eventually converted to toxic DSBs at replication forks. Overwhelming DSBs may not only saturate DSB repair, but ATR inhibition is also known to modulate DSB repair directly [33], [34] contributing to synthetic lethality observed in cells.

We also found that XRCC1 deficient cells are sensitive to cisplatin. The cisplatin sensitivity in XRCC1 deficient cells observed in our study is consistent with a recent study in HepG2 cells where cisplatin sensitivity was demonstrated following XRCC1 depletion [35]. We did not observe any potentiation of cisplatin cytotoxicity by ATR inhibitor in XRCC1 wild type cells. This is in contrast to previous preclinical studies where ATR inactivation (genetically or with inhibitors) has demonstrated increased platinum sensitivity in a panel of cell lines [22]. However, a limitation of our study is that our investigation was restricted to a few cell types only. Nevertheless, our data suggests that genetic background (such as XRCC1 status) may influence platinum sensitivity. In conclusion, we have demonstrated a synthetic lethality application for ATR inhibitors in XRCC1 deficient cells. ATR inhibition may also influence platinum sensitivity in XRCC1 deficient cells.

## References

[1] Friedberg EC (2003) DNA damage and repair. Nature 421: 436–440. 12540918 10.1038/nature01408

[2] Siddik ZH (2003) Cisplatin: mode of cytotoxic action and molecular basis of resistance. Oncogene 22: 7265–7279. 14576837 10.1038/sj.onc.1206933

[3] Rabik CA, Dolan ME (2007) Molecular mechanisms of resistance and toxicity associated with platinating agents. Cancer Treat Rev 33: 9–23. 17084534 10.1016/j.ctrv.2006.09.006 PMC1855222

[4] Nouspikel T (2009) DNA repair in mammalian cells : Nucleotide excision repair: variations on versatility. Cell Mol Life Sci 66: 994–1009. 19153657 10.1007/s00018-009-8737-y PMC11131503

[5] Shuck SC, Short EA, Turchi JJ (2008) Eukaryotic nucleotide excision repair: from understanding mechanisms to influencing biology. Cell Res 18: 64–72. 18166981 10.1038/cr.2008.2 PMC2432112

[6] Svilar D, Goellner EM, Almeida KH, Sobol RW (2011) Base excision repair and lesion-dependent subpathways for repair of oxidative DNA damage. Antioxid Redox Signal 14: 2491–2507. 20649466 10.1089/ars.2010.3466 PMC3096496

[7] Robertson AB, Klungland A, Rognes T, Leiros I (2009) DNA repair in mammalian cells: Base excision repair: the long and short of it. Cell Mol Life Sci 66: 981–993. 19153658 10.1007/s00018-009-8736-z PMC11131461

[8] Ladiges WC (2006) Mouse models of XRCC1 DNA repair polymorphisms and cancer. Oncogene 25: 1612–1619. 16550161 10.1038/sj.onc.1209370

[9] Horton JK, Watson M, Stefanick DF, Shaughnessy DT, Taylor JA, et al (2008) XRCC1 and DNA polymerase beta in cellular protection against cytotoxic DNA single-strand breaks. Cell Res 18: 48–63. 18166976 10.1038/cr.2008.7 PMC2366203

[10] Caldecott KW (2003) XRCC1 and DNA strand break repair. DNA Repair (Amst) 2: 955–969. 12967653 10.1016/s1568-7864(03)00118-6

[11] Wilson DM, 3rd, Kim D, Berquist BR, Sigurdson AJ (2011) Variation in base excision repair capacity. Mutat Res 711: 100–112. 21167187 10.1016/j.mrfmmm.2010.12.004 PMC3101302

[12] Corso G, Marrelli D, Pedrazzani C, Machado JC, Mancini S, et al (2009) Gastric cardia carcinoma is associated with the promoter -77T>C gene polymorphism of X-ray cross-complementing group 1 (XRCC1). J Gastrointest Surg 13: 2233–2238. 19662459 10.1007/s11605-009-0980-x

[13] Gossage L, Madhusudan S (2007) Cancer pharmacogenomics: role of DNA repair genetic polymorphisms in individualizing cancer therapy. Mol Diagn Ther 11: 361–380. 18078354 10.1007/BF03256260

[14] Gurubhagavatula S, Liu G, Park S, Zhou W, Su L, et al (2004) XPD and XRCC1 genetic polymorphisms are prognostic factors in advanced non-small-cell lung cancer patients treated with platinum chemotherapy. J Clin Oncol 22: 2594–2601. 15173214 10.1200/JCO.2004.08.067

[15] Kim K, Kang SB, Chung HH, Kim JW, Park NH, et al (2008) XRCC1 Arginine194Tryptophan and GGH-401Cytosine/Thymine polymorphisms are associated with response to platinum-based neoadjuvant chemotherapy in cervical cancer. Gynecol Oncol 111: 509–515. 18851872 10.1016/j.ygyno.2008.08.034

[16] Sun X, Li F, Sun N, Shukui Q, Baoan C, et al (2009) Polymorphisms in XRCC1 and XPG and response to platinum-based chemotherapy in advanced non-small cell lung cancer patients. Lung Cancer 65: 230–236. 19157633 10.1016/j.lungcan.2008.11.014

[17] Abdel-Fatah T, Sultana R, Abbotts R, Hawkes C, Seedhouse C, et al.. (2012) Clinicopathological and functional significance of XRCC1 (X-ray repair cross-complementing gene 1) expression in ovarian cancer. Int J Cancer. doi: 10.1002/ijc.27980. 10.1002/ijc.27980 23225521

[18] Cimprich KA, Cortez D (2008) ATR: an essential regulator of genome integrity. Nat Rev Mol Cell Biol 9: 616–627. 18594563 10.1038/nrm2450 PMC2663384

[19] Nam EA, Cortez D (2011) ATR signalling: more than meeting at the fork. Biochem J 436: 527–536. 21615334 10.1042/BJ20102162 PMC3678388

[20] Toledo LI, Murga M, Fernandez-Capetillo O (2011) Targeting ATR and Chk1 kinases for cancer treatment: a new model for new (and old) drugs. Mol Oncol 5: 368–373. 21820372 10.1016/j.molonc.2011.07.002 PMC3590794

[21] Chen T, Stephens PA, Middleton FK, Curtin NJ (2012) Targeting the S and G2 checkpoint to treat cancer. Drug Discov Today 17: 194–202. 22192883 10.1016/j.drudis.2011.12.009

[22] Peasland A, Wang LZ, Rowling E, Kyle S, Chen T, et al (2011) Identification and evaluation of a potent novel ATR inhibitor, NU6027, in breast and ovarian cancer cell lines. Br J Cancer 105: 372–381. 21730979 10.1038/bjc.2011.243 PMC3172902

[23] Cliby WA, Lewis KA, Lilly KK, Kaufmann SH (2002) S phase and G2 arrests induced by topoisomerase I poisons are dependent on ATR kinase function. J Biol Chem 277: 1599–1606. 11700302 10.1074/jbc.M106287200

[24] Caporali S, Falcinelli S, Starace G, Russo MT, Bonmassar E, et al (2004) DNA damage induced by temozolomide signals to both ATM and ATR: role of the mismatch repair system. Mol Pharmacol 66: 478–491. 15322239 10.1124/mol.66.3.

[25] Lord CJ, Ashworth A (2008) Targeted therapy for cancer using PARP inhibitors. Curr Opin Pharmacol 8: 363–369. 18644251 10.1016/j.coph.2008.06.016

[26] Banerjee S, Kaye SB, Ashworth A (2010) Making the best of PARP inhibitors in ovarian cancer. Nat Rev Clin Oncol 7: 508–519. 20700108 10.1038/nrclinonc.2010.116

[27] Rouleau M, Patel A, Hendzel MJ, Kaufmann SH, Poirier GG (2010) PARP inhibition: PARP1 and beyond. Nat Rev Cancer 10: 293–301. 20200537 10.1038/nrc2812 PMC2910902

[28] Berquist BR, Singh DK, Fan J, Kim D, Gillenwater E, et al (2010) Functional capacity of XRCC1 protein variants identified in DNA repair-deficient Chinese hamster ovary cell lines and the human population. Nucleic Acids Res 38: 5023–5035. 20385586 10.1093/nar/gkq193 PMC2926592

[29] Caldecott KW, Tucker JD, Thompson LH (1992) Construction of human XRCC1 minigenes that fully correct the CHO DNA repair mutant EM9. Nucleic Acids Res 20: 4575–9. 1408759 10.1093/nar/20.17.4575 PMC334187

[30] Fan J, Wilson PF, Wong HK, Urbin SS, Thompson LH, et al (2007) XRCC1 down-regulation in human cells leads to DNA-damaging agent hypersensitivity, elevated sister chromatid exchange, and reduced survival of BRCA2 mutant cells. Environ Mol Mutagen 48: 491–500. 17603793 10.1002/em.20312

[31] Sultana R, McNeill DR, Abbotts R, Mohammed MZ, Zdzienicka MZ, et al (2012) Synthetic lethal targeting of DNA double-strand break repair deficient cells by human apurinic/apyrimidinic endonuclease inhibitors. Int J Cancer 131: 2433–44. 22377908 10.1002/ijc.27512 PMC3742328

[32] Berenbaum MC (1981) Criteria for analyzing interactions between biologically active agents. Adv Cancer Res 35: 269–335. 7041539 10.1016/s0065-230x(08)60912-4

[33] Serrano MA, Li Z, Dangeti M, Musich PR, Patrick S, et al.. (2012) DNA-PK, ATM and ATR collaboratively regulate p53-RPA interaction to facilitate homologous recombination DNA repair. Oncogene. 10.1038/onc.2012.257 PMC3651755 22797063

[34] Sirbu BM, Lachmayer SJ, Wulfing V, Marten LM, Clarkson KE, et al (2012) ATR-p53 restricts homologous recombination in response to replicative stress but does not limit DNA interstrand crosslink repair in lung cancer cells. PLoS One 6: 1–10. 10.1371/journal.pone.0023053 PMC3155521 21857991

[35] Zhang R, Niu Y, Zhou Y (2010) Increase the cisplatin cytotoxicity and cisplatin-induced DNA damage in HepG2 cells by XRCC1 abrogation related mechanisms. Toxicol Lett 192: 108–14. 19853026 10.1016/j.toxlet.2009.10.012

